# Identification of Cinnabarinic Acid as a Novel Endogenous Aryl Hydrocarbon Receptor Ligand That Drives IL-22 Production

**DOI:** 10.1371/journal.pone.0087877

**Published:** 2014-02-03

**Authors:** Margaret M. Lowe, Jeff E. Mold, Bittoo Kanwar, Yong Huang, Alexander Louie, Michael P. Pollastri, Cuihua Wang, Gautam Patel, Diana G. Franks, Jennifer Schlezinger, David H. Sherr, Allen E. Silverstone, Mark E. Hahn, Joseph M. McCune

**Affiliations:** 1 Division of Experimental Medicine, Department of Medicine, University of California San Francisco, San Francisco, California, United States of America; 2 Division of Gastroenterology, Department of Pediatrics, University of California San Francisco, San Francisco, California, United States of America; 3 Drug Studies Unit, Department of Bioengineering and Therapeutic Sciences, University of California San Francisco, San Francisco, California, United States of America; 4 Department of Chemistry and Chemical Biology, Northeastern University, Boston, Massachusetts, United States of America; 5 Biology Department, Woods Hole Oceanographic Institution, Woods Hole, Massachusetts, United States of America; 6 Department of Environmental Health, School of Public Health, Boston University, Boston, Massachusetts, United States of America; 7 Department of Microbiology and Immunology, State University of New York Upstate Medical University, Syracuse, New York, United States of America; University Hospital of Heidelberg, Germany

## Abstract

The aryl hydrocarbon receptor (AHR) binds to environmental toxicants including synthetic halogenated aromatic hydrocarbons and is involved in a diverse array of biological processes. Recently, the AHR was shown to control host immunity by affecting the balance between inflammatory T cells that produce IL-17 (Th17) and IL-22 versus regulatory T cells (Treg) involved in tolerance. While environmental AHR ligands can mediate this effect, endogenous ligands are likely to be more relevant in host immune responses. We investigated downstream metabolites of tryptophan as potential AHR ligands because (1) tryptophan metabolites have been implicated in regulating the balance between Th17 and Treg cells and (2) many of the AHR ligands identified thus far are derivatives of tryptophan. We characterized the ability of tryptophan metabolites to bind and activate the AHR and to increase IL-22 production in human T cells. We report that the tryptophan metabolite, cinnabarinic acid (CA), is an AHR ligand that stimulates the differentiation of human and mouse T cells producing IL-22. We compare the IL-22-stimulating activity of CA to that of other tryptophan metabolites and define stimulation conditions that lead to CA production from immune cells. Our findings link tryptophan metabolism to AHR activation and define a novel endogenous AHR agonist with potentially broad biological functions.

## Introduction

The enzyme indole 2,3-dioxygenase (IDO) contributes to the innate and adaptive immune response in settings such as autoimmunity, microbial pathogenesis, and pregnancy [1]–[3]. IDO mediates the first, rate-limiting step in tryptophan metabolism to kynurenine and is upregulated under certain inflammatory conditions, most notably in response to interferons [4]. Its activity may affect immunity through two non-exclusive mechanisms: (a) creation of a local “amino acid starvation” response [4] and (b) generation of downstream metabolites with specific immunomodulatory or cytotoxic functions [5]. Tryptophan metabolites generated by IDO can suppress T cell activation and modulate T cell differentiation, although the mechanism of these effects remains largely unknown [6], [7]. Recent studies have shown that tryptophan metabolites can alter the balance of Treg and Th17 cells, two related populations of CD4^+^ T cells with opposing functions during immune responses [8].

Treg and Th17 cells share similar developmental pathways and may arise from a common progenitor [9]. Differentiation into a Treg or Th17 cell may be governed by the presence of inflammatory cytokines [10], retinoic acid [11], and/or activation of the aryl hydrocarbon receptor (AHR) [12], [13]. The AHR is a cytosolic transcription factor that is involved in many biological processes, including development, cellular differentiation and proliferation, xenobiotic metabolism, and the immune response [12]. To date, the best-studied AHR ligands are halogenated and polycyclic aromatic hydrocarbons such as 2,3,7,8-tetrachlorodibenzo-p-dioxin (TCDD) [14]. Only a few candidate endogenous ligands have been identified, many of which are tryptophan derivatives such as 2-(1′H-indole-3′-carbonyl)-thiazole-4-carboxylic acid methyl ester (ITE), tryptamine, indirubin, 6-formylindolo[3,2-b]carbazole (FICZ), and kynurenic acid [14], [15]. More recently it has also been reported that L-kynurenine, a proximal downstream product of IDO metabolism, activates the AHR [16]. The highly conserved nature of the AHR signaling pathway has prompted the search for additional natural ligands that can be directly linked to physiological functions and established as true endogenous ligands.

Although the AHR was initially proposed to affect Treg and Th17 development, a Th17-associated cytokine, IL-22, is even more specifically dependent upon AHR activation [13]. *Ahr* ^−/−^ mice retain the ability to generate some Th17 cells but are compromised in terms of IL-22 production [13], [17]. Human T cell differentiation also exhibits distinct requirements for the AHR: activation of the AHR in stimulated human T cells was found to inhibit Th17 differentiation and to promote the differentiation of CD4^+^ T cells that produce IL-22 [18]. We therefore used expansion of IL-22-producing cells as a screen for tryptophan metabolites downstream of IDO that might act as AHR ligands. We identified an endogenous tryptophan metabolite, cinnabarinic acid (CA), as an AHR ligand that can increase IL-22 production in both human and mouse CD4^+^ T cells.

## Materials and Methods

### Ethics Statement

Heparinized blood from healthy adult volunteers was collected under protocols approved for this study by the University of California, San Francisco Committee on Human Research (CHR), approval number 10-03852. Subjects gave written informed consent in accordance with the Declaration of Helsinki. Mouse experiments were performed in compliance with the University of California, San Francisco Institutional Animal Care and Use Committee guidelines with protocols approved for this study (Protocol AN085678). Mice were housed under specific pathogen free conditions at San Francisco General Hospital and were fed standard chow.

### Chemicals and mice

Cinnabarinic acid was synthesized utilizing a single-step reaction sequence. A suspension of 1 g 2-amino-3-hydroxybenzoic acid (1 g, 6.53 mmol) in 250 mL of methanol was stirred at 25°C for 15 min. Diacetoxyiodobenzene (4.31 g, 13.39 mmol) was added to the reaction mixture in portions. The color of the reaction mixture gradually changed from pale yellow-pink to red. Stirring was continued for 12 h at room temperature. The fine red precipitate was collected by filtration and washed with methanol, affording 0.58 g of cinnabarinic acid (29.6% yield). ^1^HNMR (400 MHz, d_6_-DMSO) δ 9.71 (s, 1H), 8.78 (s, 1H), 7.93 (d, J = 8.0 Hz, 1H), 7.75 (d, J = 8.0 Hz, 1H), 7.59 (dd, overlapped, 1H), 6.59 (s, 1H). ^13^CNMR (100.4 MHz, d_6_-DMSO) δ 178.1, 169.1, 166.3, 152.5, 150.5, 147.6, 142.4, 129.0, 128.8, 127.9, 126.2, 120.2, 104.9, 92.7. LCMS found 301.2, [M+H]+. The purity of CA was 96.9% as measured by LC/MS with UV detection; FICZ was not present as determined by single ion monitoring of the MS spectrum. FICZ was synthesized as described previously [19]–[21]. 4-fluoro-3-hydroxyanthranilic acid (4-F-3-HAA) was synthesized by Drs. Bill Todd and Barry Carpenter [22] and provided by Robert Schwarcz. 3-Hydroxyanthranilic acid (3-HAA) (>99.6% purity), quinolinic acid (QA), picolinic acid (PA), 3-hydroxykynurenine (3-HKA), kynurenic acid (KYA), L-kynurenine (L-KYN), laccase (from *T. versicolor*), and α-naphthoflavone were purchased from Sigma. All chemicals were of the highest purity commercially available—typically ≥98%. For some experiments, laccase was heat killed by incubation at 95°C for five minutes. The AHR antagonist, CH-223191 (2-methyl-2H-pyrazole-3-carboxylic acid (2-methyl-4-*o*-tolylazo-phenyl)-amide) [23], was purchased from CalBiochem. TCDD was purchased from Ultra Scientific. 1-(1-propynyl)pyrene (1-PP) was the generous gift of Cornelis Elferink (Univ. of Texas Medical Branch, Galveston, TX). C57BL/6 mice were purchased from Jackson Laboratory. *Ahr* ^−/−^ mice on a B6 background were derived from the line created by Schmidt et al [24].

### In vitro human cell culture

For assays involving total PBMCs, 3×10^5^ cells obtained by ficoll-hypaque density gradient centrifugation were plated with 5×10^4^ irradiated allogeneic cells in a 96 well U-bottom plate in 200 µL RPMI with 10% FBS. Cells were stimulated with plate-bound anti-CD3 (0.5 µg/mL in PBS, BD, SP34) and soluble anti-CD28 (0.5 µg/mL, BD) in the presence of tryptophan metabolite or DMSO control. The AHR antagonist CH-223191 (10 µM) was used to block AHR in some experiments. Metabolites and inhibitors were replenished on day 2 or 3.

For naïve CD4^+^ T cell sorting, human PBMCs from adult donors or from de-identified cord blood (MD Anderson) were stained with anti-CD3-Alexa700 (BD), anti-CD4-ECD (Invitrogen), anti-CD8-PeCy5.5 (Invitrogen), anti-CD45RA-FITC (BD), anti-CD95-APC (BD), anti-CD25-PE (BD), anti-CCR7-PeCy7 (BD), and anti-CD27-APCCy7 (eBioscience). Naïve CD4^+^ T cells were sort-purified as CD3^+^CD4^+^CD8^−^ CD45RA^+^CCR7^+^CD95^−^CD25^−^CD27^+^. 1×10^5^ cells were plated with 5–10×10^4^ irradiated allogeneic stimulators in 200 µL XVIVO-20 serum free media (Lonza) in 96 well U-bottom plates. Cells were incubated with CA or DMSO control under polarizing conditions with plate-bound 0.5 µg/mL anti-CD3 (SP34) and 0.5 µg/mL soluble anti-CD28, 10 ng/mL IL-21 (eBioscience), IL-1β, IL-23, 10 µg/mL anti-IFNγ, and 5 µg/mL anti-IL-4 and anti-IL12 blocking antibodies (R&D). Cord blood samples were instead stimulated with CD3/CD28 Dynabeads (Invitrogen) and additionally incubated with TGF-β (R&D). Cytokines, blocking antibodies, and metabolite were added again on days 2 and 4.

The optimal day for restimulation was determined to be day 5 or 6; cells were re-stimulated with phorbol 12-myristate 13-acetate (PMA)/ionomycin in the presence of brefeldin A (BFA) and stained for cytokine production Cells were stained extracellularly with anti-CD4-ECD and anti-CD8-PeCy5.5, and Aqua viability dye (Invitrogen), and fixed and permeabilized (BD Cytofix/Cytoperm). Samples were stained intracellularly with anti-CD3-Alexa700 or anti-CD3-APC-Cy7 (BD) to detect internalized CD3 on activated cells, as well as anti-IL-17-AlexaFluor647 (eBioscience), anti-IL-22-PE (R&D), and anti-IFNγ-PB (eBioscience) antibodies. All events were acquired on an LSRII (BD) and analyzed with FlowJo v7-9.3.2 (Treestar). CD4^+^ cells were gated as live, CD3^+^, CD4^+^CD8^−^ lymphocytes. The descriptor “Total events collected” represents all ungated events (including both live and dead events). At least 10,000 CD4^+^ events were analyzed per sample.

### Human regulatory T cell differentiation

Naïve CD4^+^ T cells were sorted as described in above. 200,000 naïve T cells were incubated in a 96 well U-bottom plate in 200 µL XVIVO-20 media. Cells were stimulated with CD3/CD28 Dynabeads (Invitrogen) in the presence of CA or DMSO vehicle control. Some cells were additionally treated with TGF-β (RND). Cells were refed by removing 100 µL of media and adding 100 µL of 2× metabolite/TGF-β on days 2 and 4, and were stained on day 6 for flow analysis. Cells were stained on days 6 with Aqua viability dye, anti-CD3-APCCy7, anti-CD4-ECD, anti-CD8-PeCy5.5, and anti-CD25-PeCy7. Cells were fixed and permeabilized with the FOXP3/Transcription Factor Staining Buffer Set (eBioscience) and stained intranuclearly with anti-FOXP3-APC or PE (eBioscience, clone PCH101).

### Human Treg suppressor assay

Tregs were generated as described above, in the presence of CA (10 µM) or DMSO control. On day 6 of culture, Dynabeads were removed, viable cells were quantified following propidium iodide staining on the Accuri C6 (BD). Autologous PBMCs were isolated and labeled with 5(6)-carboxyfluorescein N-hydroxysuccinimidyl ester (CFSE), and 100,000 PBMCs were plated in a 96-well U bottom plate in XVIVO-20 with varying ratios of Tregs. Cells were stimulated with plate-bound 0.5 µg/mL anti-CD3 (HIT3a) and 0.5 µg/mL soluble anti-CD28 for four days, then stained with anti-CD3, anti-CD4, and anti-CD8.

### In vitro mouse cell culture

Naïve CD4^+^ T cells were sort-purified from mouse splenocytes following depletion of non-CD4^+^ T cells with the MACS CD4^+^ T cell Isolation Kit II (Miltenyi). Cells were stained with anti-CD3-PB (BD), anti-CD4-PE (BD), anti-CD62L-PeCy7 (BD), anti-CD25-APCCy7 (eBioscience), and anti-CD45Rb (BD), and sorted as CD3^+^CD4^+^CD25^−^CD62L^+^CD45Rb^bright^. Cells were stimulated with 4 µL anti-CD3/CD28 Dynalbeads/well (Invitrogen), 10 ng/mL IL-1β (Peprotech), 20 ng/mL IL-6 (Peprotech), 10 ng/mL TGF-β (R&D), and 10 µg/mL anti-IFNγ and anti-IL-12/23 (UCSF Hybridoma Core) in 200 µL XVIVO-20 media. CA, KYA, L-KYN, FICZ, or laccase were added to individual wells; an equivalent volume of vehicle (DMSO) was added to control wells. 1-(1-propynyl)pyrene (1-PP) was used for CYP1A1 inhibition in some experiments. On day 2, cells were transferred to a 48-well plate. Cytokines, blocking antibodies, and metabolites, enzymes, or inhibitors were added at 2× concentration on days 2 and 4.

Cells were re-stimulated on day 5, which was determined to be optimal, with PMA/ionomycin in the presence of BFA for 4–6 hours and stained for cytokine production Cells were stained extracellularly with anti-CD4-QDot 605 (Invitrogen) and anti-CD8-PeCy5.5 or anti-CD8-PB (Caltag), Aqua viability dye, and intracellularly with anti-CD3-PB or anti-CD3-PeCy5 (BD), anti-IL-22-PE (eBioscience), anti-IL-17-APC (BD), and anti-IFNγ-APCCy7 (BD).

RNA was collected following restimulation with PMA and ionomycin for 5 hours in RLT lysis buffer. RNA was purified with Qiagen RNeasy columns. RNA input was standardized per experiment by Nanodrop before cDNA transcription reactions (Omniscript). Taqman primers for *Il22* (Mm00444241_m1) and *Hprt1* (Mm00446968_m1) were used to quantify cDNA transcript in reactions with Taqman Universal PCR master mix. Reactions were run in a StepOnePlus analyzer.

### Mouse regulatory T cell differentiation

Naïve CD4^+^ T cells were sorted from mouse splenocytes as described above. 200,000 cells were stimulated with 4 µL/well CD3/CD28 Dynalbeads in 96 well U bottom plates in 200 µL XVIVO-20 media in the presence of CA or DMSO vehicle control. TGF-β was added to some wells. On days 2 and 4, 100 µL of media were removed, and 100 µL of 2× cytokine/metabolite were re-added. On day 5, Dynalbeads were removed magnetically and cells were stained for flow analysis. Cells were stained with anti-CD4-QDot 605, anti-CD8-PeCy5.5, Aqua viability dye, anti-CD3-PB, and anti-CD25-APCCy7 (BD). Cells were fixed and permeabilized with the FOXP3/Transcription Factor Staining Buffer Set (eBioscience) and stained intranuclearly with anti-FOXP3-PE (eBioscience).

### AHR reporter assay

Mouse H1G1.1c3 cells (courtesy of Dr. M. Denison, UC Davis) were prepared as described previously [25], except that 60,000 cells were added to each well of a 96-well, black-sided plate in 200 µl of selective medium and incubated at 37°C for 24 hours. The medium was replaced with 100 µl of non-selective medium prior to compound application. A TCDD standard curve plate was prepared by applying vehicle (DMSO, 0.5%) or TCDD (10^−14^–10^−9^ M), with each concentration applied to 8 wells. For agonist experiments, vehicle or test compound were applied at a single concentration in each column, excluding two untreated columns. For antagonism experiments, compound application was immediately followed by dosing with either vehicle or TCDD (10^−10^ M). The plates were incubated at 33°C for 24 hours and EGFP fluorescence was read with a fluorometric plate reader (Synergy2, Biotek Instruments). Excitation and emission wavelengths were 485 nm (20 nm bandwidth) and 530 nm (25 nm bandwidth). Untreated well fluorescence was subtracted from experimental well fluorescence. Data were averaged from eight replicate wells. The gain was adjusted between experiments so that wells exposed to 10^−10^ M TCDD wells produced 15,000 RFUs per well; subsequent plates in the experiment were analyzed with that gain setting.

The specificity of the fluorescence measured in the H1G1 cells treated with CA, HAA, and tryptamine was determined by concurrently treating Hepa-1 cells (the parent line of the H1G1 cell line) with CA, HAA and tryptamine at the same concentrations. Treatment, incubation, and analysis were carried out as above. Fluorescence measured in Hepa-1 cells was subtracted from the fluorescence measured in H1G1 cells treated with the same concentration of CA, HAA or tryptamine to correct for background fluorescence.

### In vitro AHR competitive-binding assay

Human AHR protein was synthesized from an AHR expression construct (pSporthAHR2, courtesy of Dr. C. Bradfield, U. of Wisconsin, Madison, WI) [26] using a TnT-Quick Coupled Reticulocyte Lysate System (Promega). Competition with 2,3,7,8-tetrachloro[1,6-^3^H]dibenzo-p-dioxin ([^3^H]TCDD; 35 Ci/mmol; Chemsyn Science Laboratories) for binding to human AHR was measured by velocity sedimentation on sucrose gradients in a vertical tube rotor [27]. Single TnT reactions (50 µl) were diluted 1∶1 with MEEDMG buffer and incubated overnight at 4°C with [^3^H]TCDD (2 nM) ± competitors (dissolved in DMSO) before application to sucrose gradients. Nonspecific binding was determined by using TnT reactions containing an empty vector.

### CYP1A1 induction

Zebrafish embryos [TL strain; 48 or 72 hours post fertilization (hpf)] were exposed to CA (100 µM) or DMSO for 6 hours. Following exposure, three replicate groups of 20 embryos from each treatment group were frozen in liquid nitrogen. In one experiment, 72-hpf embryos exposed for 6 hr to CA or DMSO were subsequently held in clean water and sampled at 96 hpf. Total RNA was isolated from frozen embryos using RNA STAT-60 (Tel-Test B, Inc.). cDNA was synthesized from 2 µg of total RNA using Omniscript reverse transcriptase (Qiagen). Real-time RT-PCR for *cyp1a* and *beta-actin* was performed using the iQ SYBR Green Supermix (Bio-Rad) in an iCycler iQ Real-Time PCR Detection System (Bio-Rad) as described previously [28]. Fold induction of *cyp1a* by CA was calculated using the ΔΔC_T_ method [29].

Human PBMCs were isolated by ficoll hypaque density gradient centrifugation and plated in 48-well plates at a density between 0.5–2×10^6^ cells per well in 1 mL of RPMI. Cells were stimulated with 1 µg/mL PHA in the presence of tryptophan metabolite or DMSO for 8–20 hours and harvested in RLT lysis buffer. RNA was purified with Qiagen RNeasy columns. RNA input was standardized per experiment by Nanodrop before cDNA transcription reactions (Omniscript). Taqman primers for *CYP1A1* (Hs00153120_m1) and *HPRT1* (Hs99999909_m1) were used to quantify cDNA transcript in reactions with Taqman Universal PCR master mix. Reactions were run in a StepOnePlus analyzer.

Mouse lymphocytes were isolated from brachial, axillary, and inguinal lymph nodes, and were then plated in 96 well plates at a density of 1×10^6^ cells/well in 200 µL of RPMI, cultured for 4 hours, and lysed in RLT lysis buffer. RNA isolation and RT-PCR was handled as with human cells, except with Taqman mouse *Cyp1a1* (Mm00487217_m1) and *Hprt1* (Mm00446968_m1) primers.

### CYP1A1 inhibition

1.8 pmol of human CYP1A1 + P450 Reductase Supersomes (BD) were used per reaction in the luminescent-based P450-Glo CYP1A1 Assay (Promega). Briefly, CYP1A1 Supersomes, luciferin-CEE substrate, and test compound were equilibrated in white opaque 96-well plates (Pierce) for 10 minutes at 37°C per kit protocol. NADPH Regenerating Solution (Promega) was added, and reactions were terminated after 15 minutes at 37°C by addition of luciferase detection reagent. Luminescence was read with the SpectraMax M2 microplate reader using SoftMax Pro software (Molecular Devices) and averaged over 6–8 reads per well.

### CA detection

PBMCs were plated at a concentration of 2.5–5×10^6^ cells/well in 200 µL of RPMI. Cells were left unstimulated or incubated with 50 µg/mL LPS, 10 ng/mL PMA, 100 ng/mL IFNγ, or 1–5 µg/mL concanavalin A (conA) for 16 hours. Supernatants were frozen for detection of CA by LC/MS/MS.

### LC/MS/MS

CA was measured by liquid chromatography-tandem mass spectrometry (LC/MS/MS). Samples (20 µl) were mixed with 100 µl of internal standard, piroxicam (100 ng/ml) in acetonitrile, vortexed for 1 min, and centrifuged at 3000 rpm for 10 min. The supernatant was transferred to an autosampler vial and 8 µl was injected to the LC/MS/MS system. The standard curve was generated by serial diluting CA standard solution in water. The mass detector was an API 5000 triple quadrapole (Applied Biosystems), equipped with a Turbo Ion Spray source. The system was set in positive ionization mode. The ion spray voltage was 5500 V and the source temperature was 600°C. The values for CAD, CUR, GS1, and GS2 were 6, 15, 55, and 75 respectively. The multiple reaction monitor was set at 301.1 – 265.0 *m/z* for CA and 332.0 – 94.9 *m/z* for piroxicam. A Shimadzu system was used for the HPLC, consisting of a pump, solvent degasser, autosampler and column oven, which was set to 30°C. The mobile phase, consisting of 40% acetonitrile, 0.1% trifluoroacetic acid containing 5 mM ammonium acetate, was pumped through a Synergi Polar RP (4.6×75 mm, 4 µm particle size) column with a flow rate of 1.0 ml/min. Data were acquired and processed by Analyst 1.5.1 software.

The limit of quantification (LOQ) and limit of detection (LOD) for CA were 7.81 ng/ml and 3 ng/ml, respectively. The tryptophan, L-KYN, and 3-HAA levels were measured in API-5000 with a similar method as reported before [30]. CA values below LOQ were treated as ½ LOQ for statistical analysis [31].

### Statistical analysis

Statistical tests used to analyze data are denoted individually within figure legends (GraphPad Prism v.4.0c). P values <0.05 were considered statistically significant.

## Results

### 3-Hydroxyanthranilic acid (3-HAA) increases the frequency of IL-22-expressing CD4^+^ T cells in vitro

**Figure 1:**
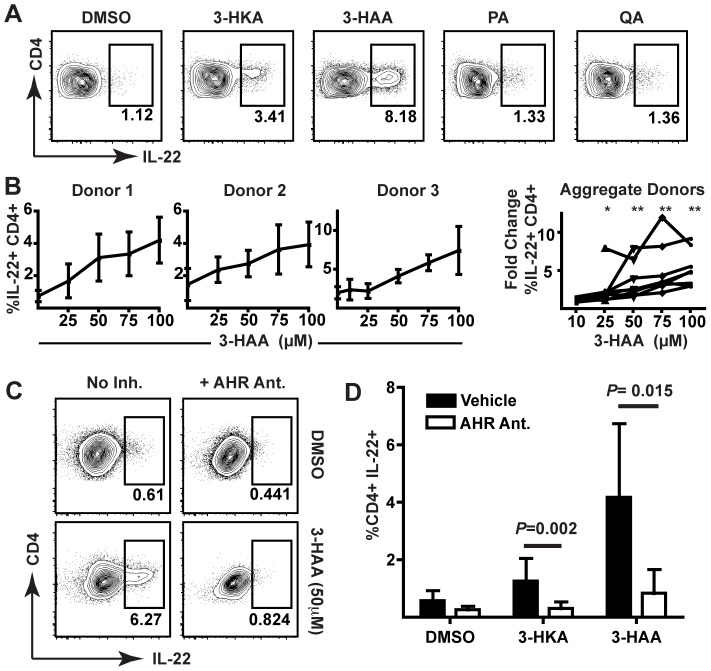
3-HKA and 3-HAA promote IL-22 expression in stimulated human CD4^+^ T cells. (A) Flow cytometric analysis of CD4^+^ T cells following stimulation of human PBMCs in the presence of 100 µM 3-HKA, 3-HAA, PA, or QA for six days. Data represent at least three independent experiments. (B) Flow cytometric analysis of CD4^+^ T cells from individual and aggregate donors following stimulation of PBMCs in the presence of increasing concentrations of 3-HAA (µM) for six days. Individual donor data are pooled from at least three independent experiments. Error bars indicate SD. Fold change in IL-22 expression versus vehicle control is statistically different from 1 (Wilcoxon signed rank test; *, p = 0.0312; **, p = 0.0078; N = 8 donors). (C) Flow cytometric analysis of CD4^+^ T cells following stimulation of human PBMCs in the presence of 3-HAA +/− the AHR antagonist, CH-223191. Data are representative of at least three independent experiments. (D) Comparison of IL-22 production in CD4^+^ T cells following stimulation of human PBMCs in the presence of DMSO, 3-HKA (50 µM), or 3-HAA (50 µM), with or without an AHR antagonist, N = 6. P values were calculated by Mann-Whitney. Error bars indicate SD.

**Figure 2:**
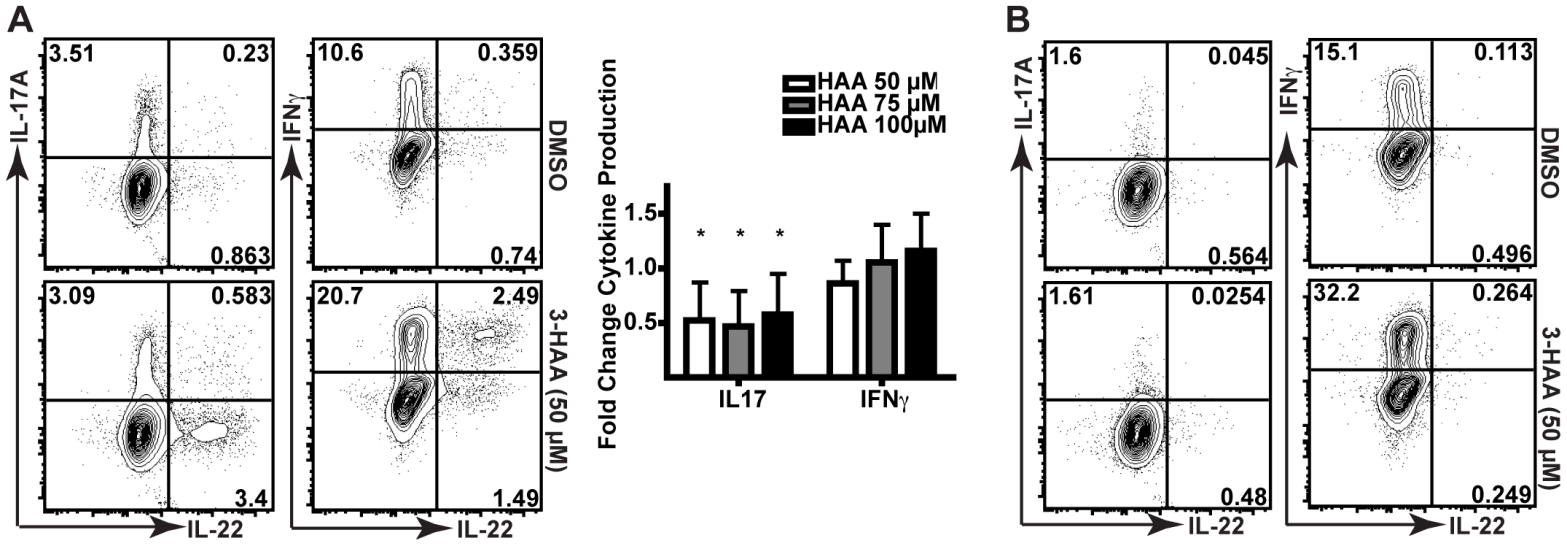
IL-22^+^ upregulation occurs in CD4^+^8^-^ T cells that are IL-17 negative but express IFNγ. (A) Cytokine production for live CD3^+^CD4^+^CD8^−^ T cells from human PBMC cultures that were stimulated with anti-CD3 and anti-CD28 antibodies and allogeneic APCs for six days with DMSO or 50 µM 3-HAA. Panel (right) depicts fold-changes in 9 donors relative to DMSO. *, p<0.05 indicates data analyzed by Wilcoxon signed rank test is statistically different than 1. (B) Cytokine production for live CD3^+^CD8^+^CD4^−^ T cells in PBMC cultures, stimulated as above with DMSO or 3-HAA (50 µM). Data are representative of three experiments.

Human PBMCs were stimulated with antibodies against CD3 and CD28 *in vitro* in the presence of different tryptophan metabolites, including 3-hydroxykynurenine (3-HKA), 3-hydroxyanthranilic acid (3-HAA), picolinic acid (PA), and quinolinic acid (QA). 3-HKA and 3-HAA, but not the downstream metabolites PA or QA, were able to promote IL-22 production in stimulated CD4^+^ T cells (Fig. 1A). Though donors differed in the frequency of IL-22^+^ cells detected following 3-HAA exposure, 3-HAA was able to promote a 2-fold or greater expansion of IL-22-producing cells in each (Fig. 1B). Statistically significant expansion of these cells was seen beginning at 25 µM 3-HAA (Fig. 1B, aggregate donors). Additional analysis revealed that this increase also occurred within total events collected and was therefore not likely an artifact of preferential cell death (Fig. S1). To determine whether the expansion of IL-22-producing cells within this population was AHR-dependent, we stimulated human PBMCs in the presence or absence of a potent AHR antagonist (CH-223191) and observed that CH-223191 abolished the increase in IL-22 production caused by 3-HAA (Fig. 1, C and D). Within PBMC cultures stimulated in the presence of 3-HAA, upregulation of IL-22 was only seen in CD4^+^ T cells (Fig. 2A) and not in CD8^+^ T cells (Fig. 2B). These IL22^+^ CD4^+^ T cells frequently co-expressed IFNγ but not IL-17A; interestingly, IL-22^+^IL17^−^ CD4^+^ T cells identified in humans also frequently co-express IFNγ (Fig. 2A) [18]. 3-HAA suppressed IL-17 production within CD4^+^ T cells, as has been previously reported [30].

### 3-HKA and 3-HAA are not AHR ligands but may be precursors to an AHR ligand

**Figure 3:**
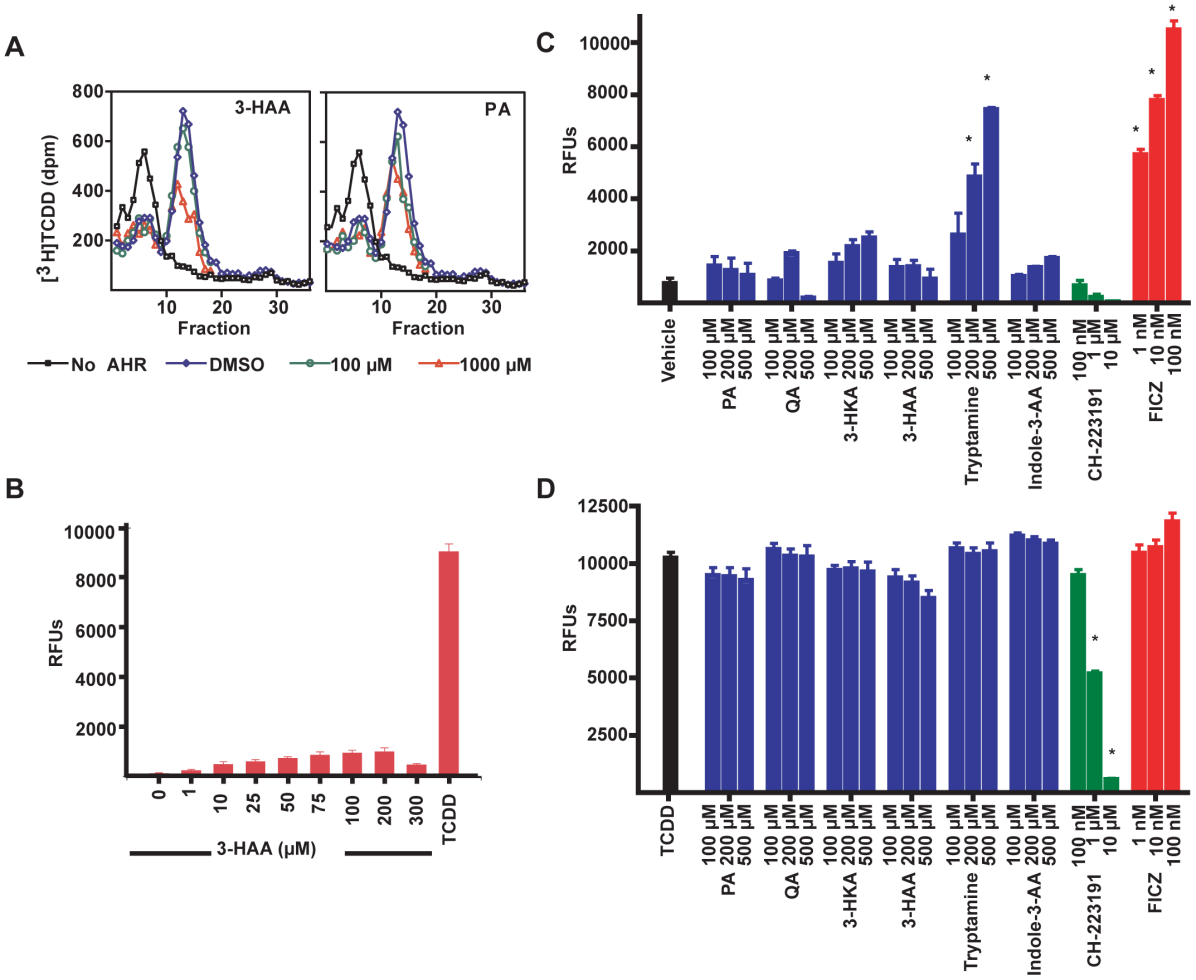
3-HAA fails to bind or activate the AHR. (A) Displacement of [^3^H]TCDD from the human AHR in the presence of 3-HAA or PA. Data for 100 µM concentrations were from 2–4 experiments; data for 1000 µM concentrations were from one or two experiments. (B) Fluorescence of an AHR-responsive reporter construct measured after incubation of a stably transfected murine hepatoma cell line in the presence of increasing concentrations of 3-HAA. TCDD (5*10^−11^ M) is a positive control. Error bars are SD. N = 6. (C) Fluorescence of the AHR-reporter construct following incubation of transfected cells with 3-HKA, PA, or QA. Tryptamine and FICZ are positive controls. (D) Inhibition of TCDD (10^−10^-M)-induced activation of the AHR reporter construct. CH-223191 is a positive control. *, p<0.05 (ANOVA, Scheffe's). Error bars are SD.

The above studies identified 3-HAA and, to a lesser extent, 3-HKA as potential ligands of the AHR. The ability of these metabolites as well as that of PA to bind and activate the AHR was assayed using two techniques. First, we determined whether each compound could bind to AHR, using a well-established assay that measures the ability of compounds to displace [^3^H]TCDD from full-length human AHR protein expressed *in vitro*. Modest binding of 3-HAA and PA was evident, but only at very high concentrations (1000 µM, Fig. 3A). We also measured AHR activation and antagonism within a reporter cell line expressing AHR responsive elements upstream of a GFP reporter. While a slight increase in AHR activity measured by fluorescence was found using 3-HAA as an agonist (Fig. 3B), this failed to reach significance, and none of the other metabolites downstream of 3-HKA tested were found to have activity as either agonists or antagonists (Fig. 3, C and D). These results suggested that 3-HAA might be an upstream precursor of an endogenous AHR ligand.

### Inhibition of 3-HAA metabolism increases IL-22 production

**Figure 4:**
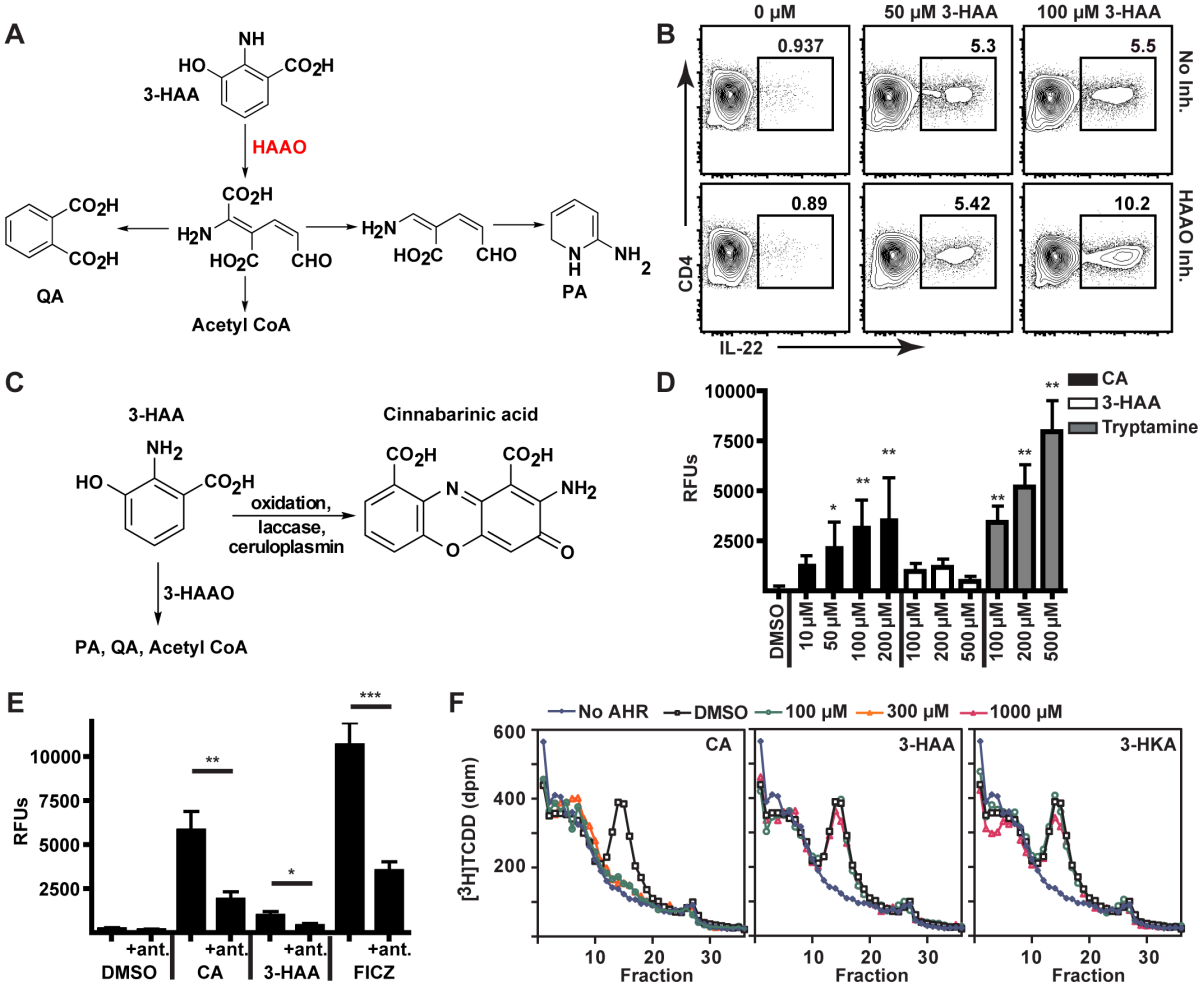
CA is an endogenous AHR agonist generated by oxidative dimerization of 3-HAA. (A) Metabolic pathways downstream of 3-HAA catalyzed by the enzyme, 3-hydroxyanthranilate 3,4-dioxygenase (HAAO), including intermediates upstream of PA and QA. (B) Flow cytometric analysis of CD4^+^ T cells in human PBMCs stimulated in the presence of varying concentrations of 3-HAA (50 µM or 100 µM) with or without the HAAO inhibitor, 4-F-3-HAA (50 µM). Data are representative of three experiments. (C) An alternative pathway of 3-HAA metabolism can generate CA either by non-enzymatic (oxidation) or enzymatic (such as laccase or ceruloplasmin) processes. (D) Fluorescence [measured in relative fluorescence units (RFUs)] of the AHR-responsive reporter construct in cells incubated with varying concentrations of 3-HAA or CA. Tryptamine was a positive control. *, p<0.05 (ANOVA, Scheffe's). Error bars represent SD. (E) Fluorescence of an AHR-responsive reporter construct measured following incubation of H1G1.1c3 cells with DMSO control, CA (200 µM), 3-HAA (200 µM), or the positive control FICZ (100 nM), with or without the AHR antagonist CH-223191 (50 µM). *, p<0.01. **, p<0.001. ***, p<0.0001. (2-way ANOVA, Bonferroni's post-test). Error bars are SD. (F) [^3^H]TCDD displacement from *in vitro* translated human AHR protein by incubation with varying concentrations of CA, 3-HAA, or 3-HKA. Data are representative of four (CA and 3-HAA) or two (3-HKA) independent experiments.

Because neither PA nor QA, the primary metabolites downstream of 3-HAA, was found to have any impact on IL-22 production, we thought it unlikely that either was an AHR ligand. The enzyme upstream of PA and QA, 3-hydroxyanthranilate 3,4-dioxygenase (HAAO), is also expressed by monocytes and macrophages, the same cell populations that express IDO under inflammatory conditions [32]. To determine whether downstream intermediates of 3-HAA generated through HAAO were acting as AHR ligands, the specific inhibitor, 4-F-3-HAA, was used to block HAAO activity (Fig. 4, A and B). Contrary to this hypothesis, HAAO inhibition within stimulated PBMC cultures did not block the ability of 3-HAA to upregulate IL-22 production; at higher concentrations of 3-HAA, HAAO inhibition increased IL-22 production (Fig. 4B). This suggested that an AHR ligand might arise from an alternative pathway of 3-HAA metabolism, which would be favored during HAAO inhibition and would result in increased IL-22 production.

### Identification of CA as an AHR ligand downstream of 3-HAA

3-HAA is susceptible to oxidation, resulting in the formation of CA through 3-HAA dimerization, both in solution [33] and in cell culture (Fig. 4C) [19]. As a tricyclic aromatic compound, the structural features of CA resemble those of some AHR ligands. Additionally, CA has potent effects on thymocyte maturation [19], similar to those observed in cultured thymocytes treated with the AHR agonist, TCDD [34]. To test the hypothesis that it is an AHR ligand, CA was synthesized and tested for its ability to bind to and activate the AHR *in vitro*. CA induced GFP in the AHR reporter cell line (Fig. 4D) and the response to CA was inhibited by the AHR antagonist, CH-223191 (Fig. 4E). CA also displaced [^3^H]TCDD from *in vitro*-expressed human AHR and was effective at much lower concentrations than those required for 3-HAA and 3-HKA (Fig. 4F).

### CA upregulates IL-22 within CD4^+^ T cells in an AHR-dependent manner

**Figure 5:**
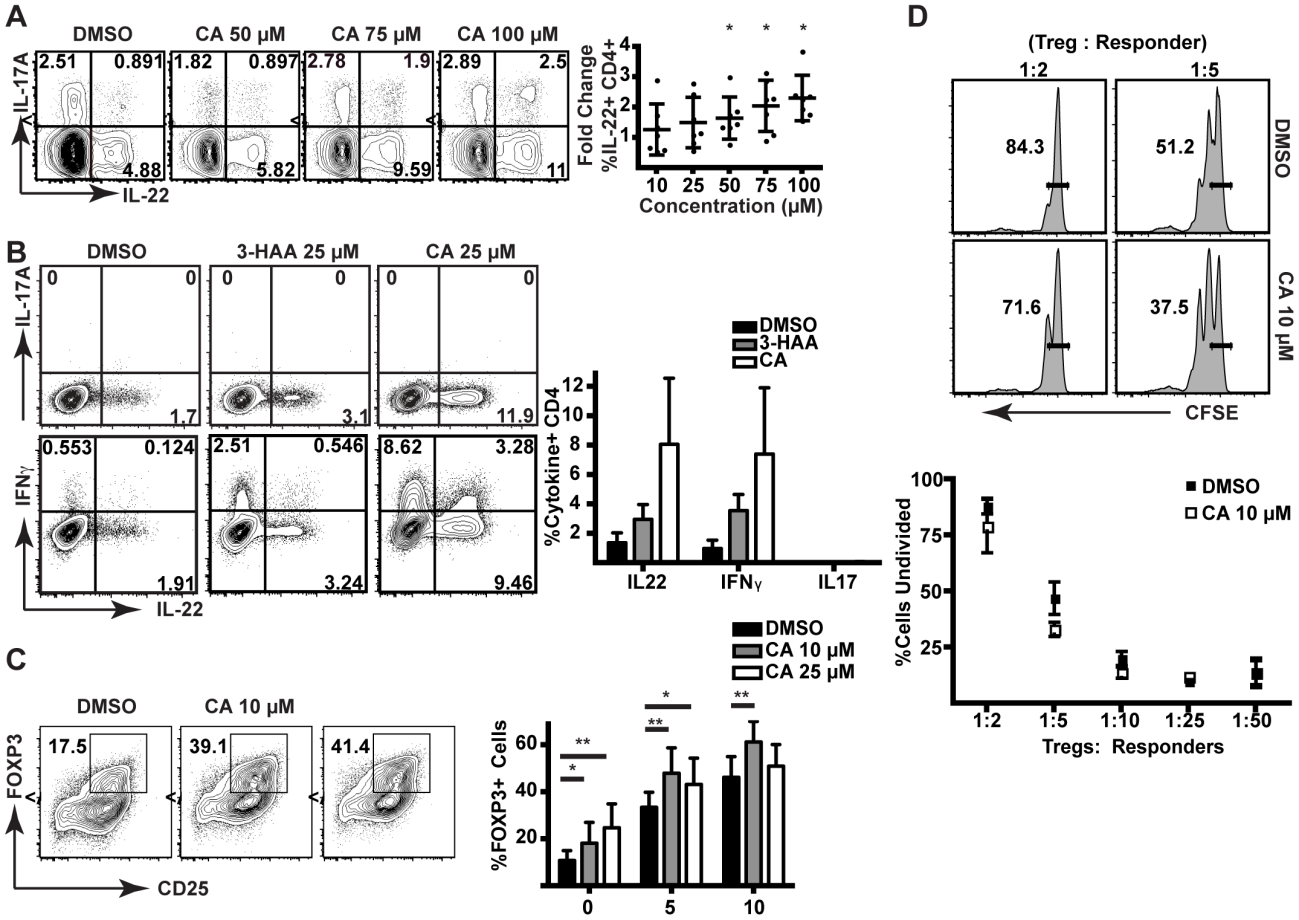
CA increases the differentiation of IL-22^+^ human CD4^+^ T cells *in vitro*. (A) Flow cytometric analysis of CD4^+^ T cells from human PBMCs stimulated in the presence of DMSO or increasing doses of CA (left). Fold change in IL-22 production in CD4^+^ T cells from human PBMCs from multiple donors stimulated in the presence of CA versus DMSO control (right panel). Data were analyzed by Wilcoxon signed rank test for significant deviation from a theoretical median of 1.000. *p<0.05. (B) Flow cytometric analysis of sorted naïve human CD4^+^ T cells stimulated under polarizing conditions (with IL-21, IL-1β, IL-23, anti-IFNγ, anti-IL-4 and anti-IL12) with DMSO, 3-HAA (25 µM), or CA (25 µM). Data on IL-22, IFNγ, and IL-17 production from three independent experiments are shown in the panel on the right. Error bars are SD. (C) Flow cytometric analysis of FOXP3 staining following stimulation of sorted naïve human CD4^+^ T cells in the presence of DMSO or CA without addition of TGF-β. Quantification of %FOXP3^+^CD25^+^ T cells of CD4^+^ cells from six donors in seven independent experiments (right panel) with increasing concentrations of TGF-β. Data were analyzed by two-way ANOVA and Holm-Sidak multiple comparisons test. *, p<0.05. **, p<0.01. Error bars are SD. (D) Flow cytometric analysis of CFSE-labeled responder CD8^+^ T cells incubated with autologous Tregs generated in the presence of CA or DMSO control. Data are representative of three independent experiments.

Treatment of adult human PBMCs with CA resulted in upregulation of IL-22, but not IL-17, within human CD4^+^ T cells in a dose-dependent manner (Fig. 5A, S2). To determine whether CA was acting on T cells directly, adult human and mouse naïve CD4^+^ T cells were sort-purified and incubated with tryptophan metabolites under polarizing conditions (see Methods for details). In the presence of 25 µM CA, human naïve CD4^+^ T cells upregulated the production of IL-22 and IFN-γ to a greater extent than those incubated with an equivalent concentration of 3-HAA (Fig. 5B, S3). Given the role of the AHR in regulating Treg differentiation [12], we additionally tested whether CA was able to affect the differentiation of Tregs as measured by expression of FOXP3 and functional activity. An expansion of FOXP3^+^ cells was seen in human naïve CD4^+^ T cells stimulated in the presence of CA (Fig. 5C, S5). However, the T cells exposed to CA were not more suppressive than DMSO control cells, despite the greater abundance of FOXP3^+^ cells (Fig. 5D).

**Figure 6:**
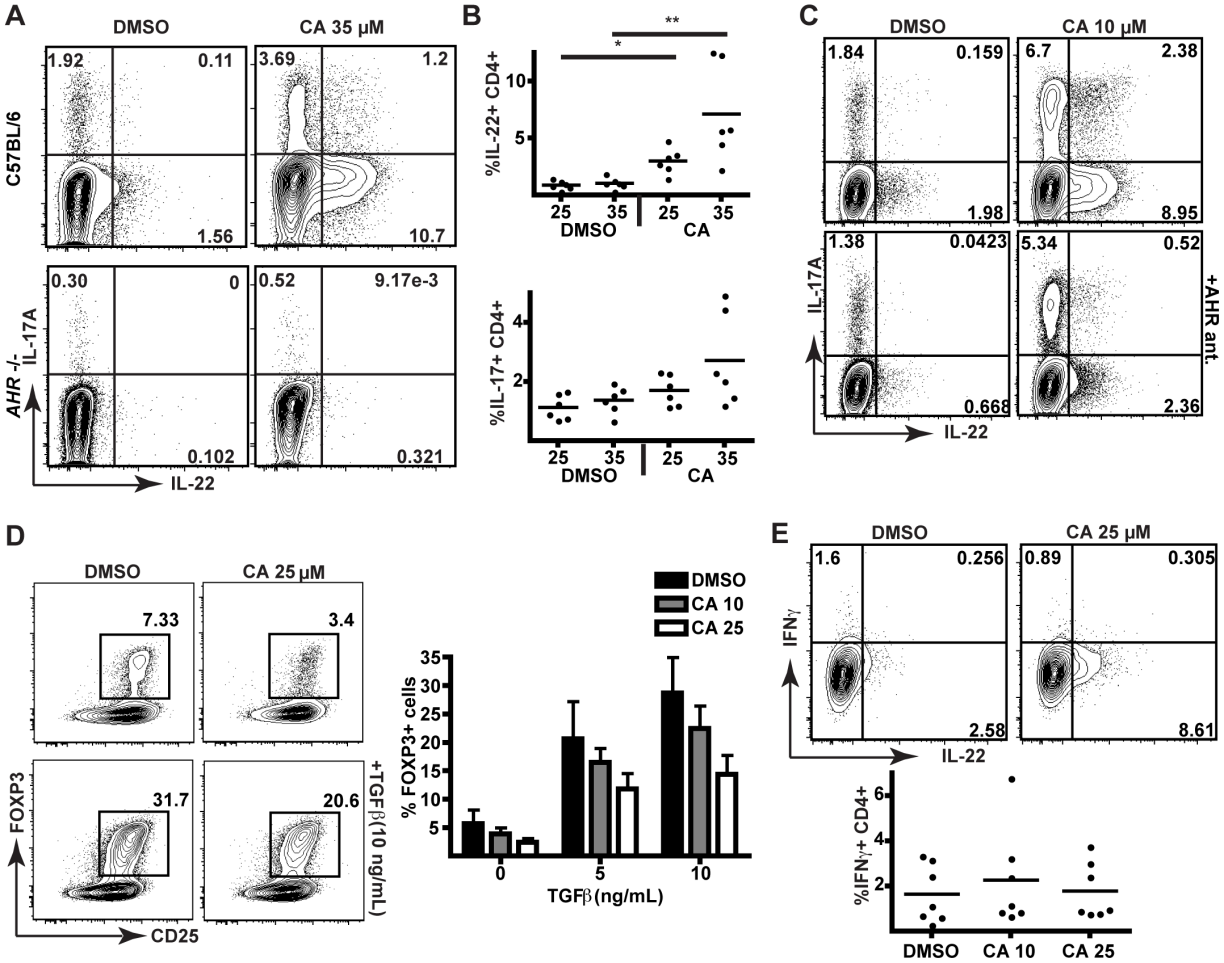
CA increases the differentiation of IL-22^+^ mouse CD4^+^ T cells *in vitro*. (A) Flow cytometric analysis of IL-17 and IL-22 production in sorted naïve mouse CD4^+^ T cells from C57BL/6 (*Ahr* ^+/+^) or *Ahr^−/−^* mice stimulated under polarizing conditions (with IL-1β, IL-6, TGF-β, anti-IFNγ, and anti-IL12/23) in the presence of DMSO or CA (35 µM). (B) Flow cytometric data for IL-22 (top panel) and IL-17 (bottom panel) production from six independent experiments in C57BL/6 mice were analyzed by Kruskal-Wallis ANOVA and Dunn's Multiple Comparison test. *, p<0.05. **, p<0.01. DMSO controls for the 25 and 35 µM CA experiments are shown separately. (C) Flow cytometric analysis of IL-17 and IL-22 production in C57BL/6 mouse naïve CD4^+^ T cells stimulated under polarizing conditions in the presence of DMSO or CA, with or without AHR antagonist, CH-223191 (10 µM). Data are representative of three independent experiments. (D) Flow cytometric analysis of FOXP3 expression in sorted naïve wild-type mouse CD4^+^ T cells stimulated with increasing concentrations of TGFβ. Quantification of %FOXP3^+^CD25^+^ T cells of CD4^+^ cells from four independent experiments is shown in the panel on the right. Error bars are SD. (E) Flow cytometric analysis of IFN-γ production in sorted naïve wild-type mouse CD4^+^ T cells stimulated under polarizing conditions (as in panel A) in the presence of DMSO or CA. Data are from seven independent experiments (right) were analyzed by Friedman ANOVA; no statistically significant differences were found.

CA treatment of mouse naïve CD4^+^ T cells also resulted in significant expansion of IL-22^+^ cells and a trend towards increased IL-17^+^ cells (Fig. 6A, 6B, S5). However, when sort-purified naïve CD4^+^ T cells isolated from *Ahr* ^−/−^ mice were treated with CA under polarizing conditions, no expansion of IL-22 producing cells occurred (Fig. 6A), demonstrating that this effect was AHR-dependent. In addition, an AHR antagonist inhibited CA-driven IL-22 production in CD4^+^ T cells from C57BL/6 (*Ahr* ^+/+^) mice (Fig. 6C), further supporting the conclusion that CA acts via AHR to increase production of IL-22. Unlike in human cells, incubation of mouse naïve cells with CA suppressed generation of FOXP3^+^ cells (Fig. 6D, S6). Nevertheless, the lack of suppressive activity in human cells treated with CA indicates that this phenotypic difference between species may not result in a change in function. In contrast, the expansion of IFN-γ producing cells seen in human cells was not observed within mouse naïve cell cultures (Fig. 6E).

### Induction of CYP1A1 and IL-22 and comparison of CA to other tryptophan metabolites

**Figure 7:**
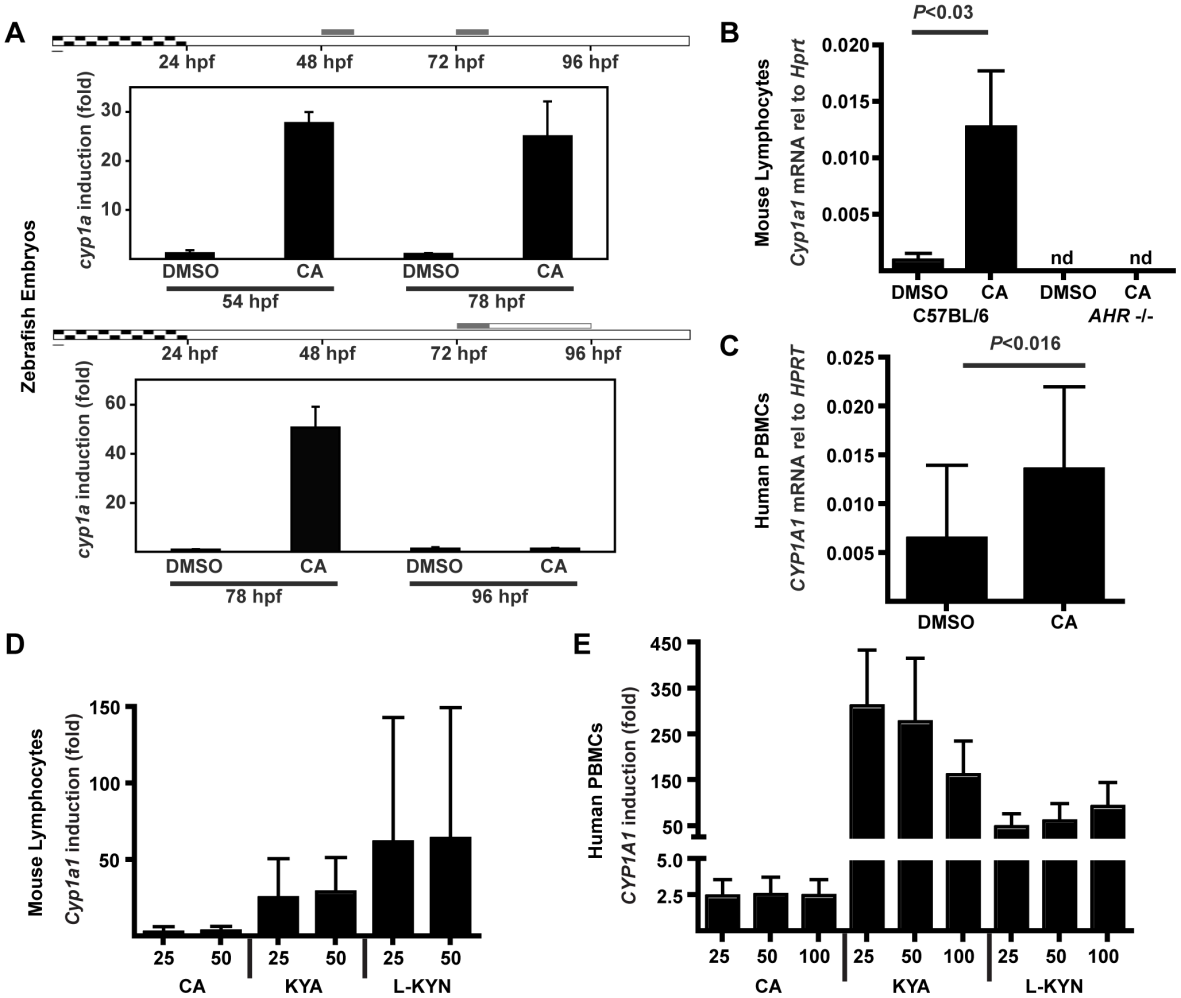
CA induces expression of AHR-responsive gene *Cyp1a1*, but with reduced efficacy versus other tryptophan metabolites. (A) Induction of *cyp1a in vivo* as measured by qRT-PCR. Zebrafish embryos at 48 or 72 hours post fertilization (hpf) were exposed to CA (100 µM) for 6 hours, and either sampled immediately (54 or 78 hpf; top panel) or placed in clean water and sampled at 96 hpf (24 hours after beginning of exposure; bottom panel). *Cyp1a* mRNA was normalized to *beta-actin* and to the average DMSO value. (DMSO values for 78 and 96-hpf embryos were similar). Values represent fold-change in CA-treated versus DMSO control embryos; each panel represents an experiment sampling three replicate groups of twenty embryos per group. (B) Induction of *Cyp1a1* in wild-type (*Ahr* ^+/+^) or *Ahr* ^−/−^ mouse total lymphocytes incubated with CA (50 µM) in RPMI for 4 hours. *Cyp1a1* was measured by qRT-PCR and normalized to *Hprt*. Error bars represent SD. P values were calculated with the Mann-Whitney test from lymphocyte cultures from three individual mice. (C) Induction of *CYP1A1* PHA-stimulated human total PBMCs after 12–20 hours of incubation with CA (50 µM) in RPMI. *CYP1A1* was measured by qRT-PCR and normalized to *HPRT*. Data shown are pooled experiments from six individual donors. Error bars are SD. P values were calculated with the Mann-Whitney test. (D, E) Induction of mouse *Cyp1a1* (D) and human *CYP1A1* (E) measured by qRT-PCR relative to *Hprt* or *HPRT*. Mouse lymphocytes (D) and PHA-stimulated human PBMCs (E) were incubated with CA, KYA, L-KYN. Values are pooled from at least three independent experiments and represent fold-change versus averaged DMSO control. Metabolite concentrations are in µM. Error bars represent SD.

CA was additionally tested for its ability to induce the well-known AHR responsive gene *CYP1A1 in vivo* and *in vitro*. In zebrafish embryos, a model vertebrate *in vivo* system, *Cyp1a* was strongly induced after 6 hours of exposure to CA (Fig. 7A, upper panel); this effect was lost 18 hours after washout of CA (Fig. 7A, lower panel). The degree of induction (25- to 50- fold) was comparable to that produced by FICZ under similar experimental conditions (∼30-fold) [35]. CA also caused statistically significant induction of *Cyp1a1* in mouse lymphocytes *in vitro* (Fig. 7B). No *Cyp1a1* induction was detected in lymphocytes derived from *Ahr^−/−^* mice (Fig. 7B), demonstrating that CA was acting through the AHR. Human PBMCs also exhibited a statistically significant, although modest, induction of *CYP1A1* (Fig. 7C).

**Figure 8:**
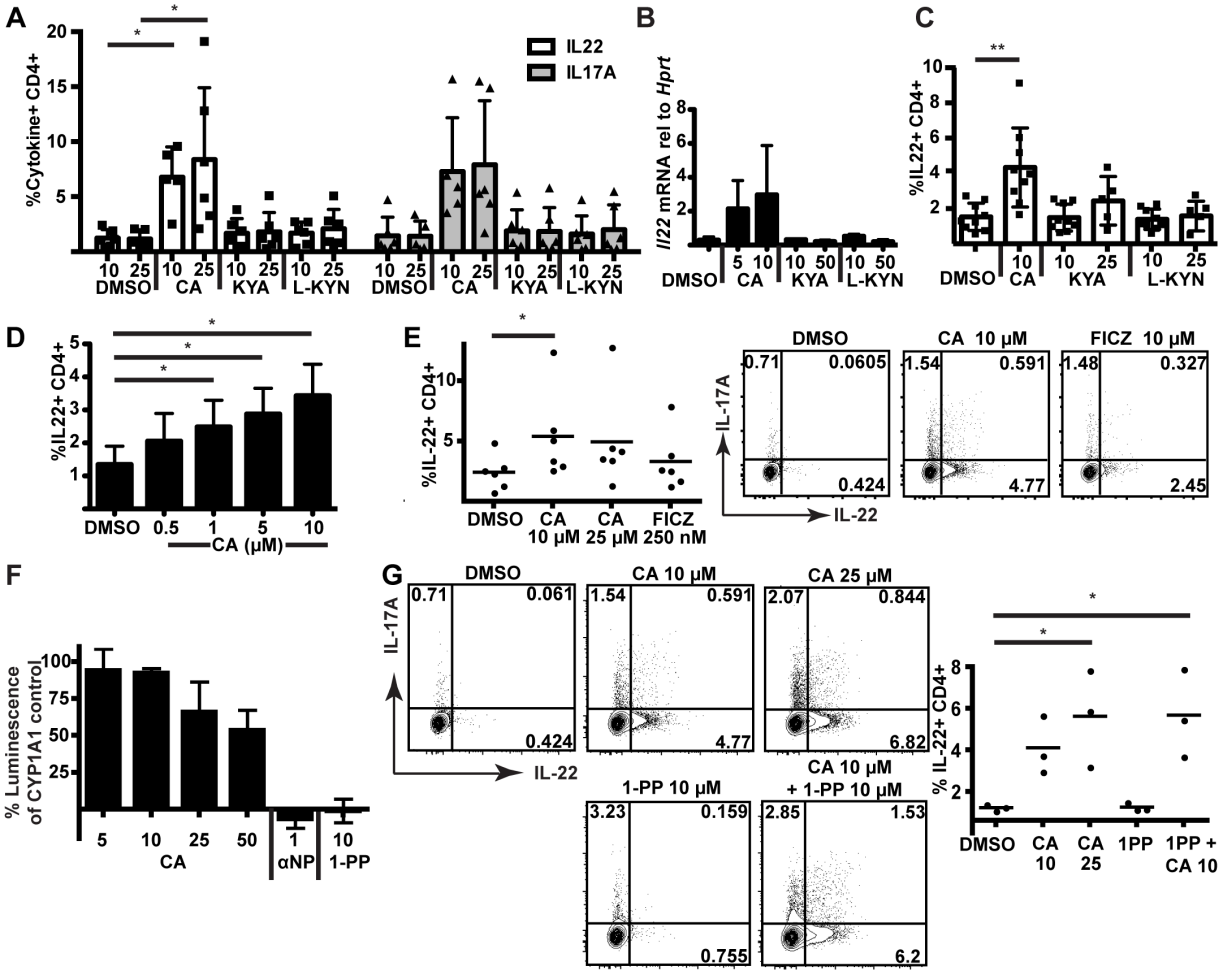
CA increases IL-22 and IL-17 production more efficiently than other tryptophan metabolites. (A) Flow cytometric analysis of mouse naïve CD4^+^ T cells for IL-22 and IL-17 production following stimulation under polarizing conditions (IL-1β, IL-6, TGF-β, anti-IFNγ, and anti-IL12/23) with DMSO, CA, L-KYN, or KYA (concentrations in µM). Data from six independent experiments were analyzed by analyzed by Kruskal-Wallis ANOVA and Dunn's Multiple Comparison test. **, p<0.01. (B) qRT-PCR data from mouse naïve CD4^+^ T cells stimulated under polarizing conditions for the expression of *Il22* relative to *Hprt*. Data are from three independent experiments; concentrations are in µM. (C) Flow cytometric analysis of human cord blood naïve CD4^+^ T cells stimulated under polarizing conditions (IL-1β, IL-6, IL-23, TGF-β, anti-IFNγ, and anti-IL4) with DMSO, CA, L-KYN, or KYA (concentrations in µM). Data from eight experiments were analyzed by repeated measures ANOVA and Dunnett's Multiple Comparisons Test. *, P<0.05. (D) Flow cytometric analysis of cord blood naïve CD4^+^ T cells stimulated under polarizing conditions with DMSO or CA. Data from four experiments were analyzed by repeated measures ANOVA and Dunnett's Multiple Comparisons Test. *, p<0.05. (E) Flow cytometric analysis of mouse naïve CD4^+^ T cells stimulated under polarizing conditions with DMSO, CA, or FICZ. Data from six independent experiments were analyzed by the Friedman test and Dunn's Multiple Comparison test. *, p<0.05. Error bars represent SD. (Right) Data testing equivalent concentrations of FICZ and CA are representative of three independent experiments. (F) Inhibition of CYP1A1 activity measured by luminescence of CYP1A1-driven metabolism of luciferin-CEE. Luminescence is a percentage of the CYP1A1 only control with background readings subtracted. Concentrations are in µM. Values are averaged from three replicate wells. Data are representative of three independent experiments. (G) Flow cytometric analysis of mouse naïve CD4^+^ T cells stimulated under polarizing conditions in the presence of the CYP1A1 inhibitor, 1-PP. Data three independent experiments were analyzed by one-way ANOVA and Dunnett's Multiple Comparison test. *, p<0.05.

CA was then compared to other tryptophan metabolites, kynurenic acid (KYA) and L-kynurenine (L-KYN), which have recently been identified as AHR agonists [15], [16]. In both mouse (Fig. 7D) and human (Fig. 7E) lymphocytes, CA induced *Cyp1a1* but was less effective than these other tryptophan metabolites. Surprisingly, however, CA was much more effective at increasing IL-22 production in these cells; neither KYA nor L-KYN was able to drive IL-22 or IL-17 protein production or increase *Il22* mRNA within mouse naïve T cells at concentrations effective at inducing *Cyp1a1* (Fig. 8A and B). Likewise, KYA and L-KYN did not increase IL-22 as effectively as CA in human naïve T cells (Fig. 8C, S7). To test the minimal effective dose for IL-22 induction, we differentiated human naïve T cells in the presence of decreasing doses of CA. Concentrations as low as 1 µM were found to significantly increase IL-22 production (Fig. 8D, S8).

Recently, it has been reported that weak AHR agonists may be capable of inducing AHR-responsive genes indirectly by inhibiting the CYP1A1-mediated metabolism of FICZ, a tryptophan photoproduct capable of activating the AHR [36]. To test whether CA was indirectly increasing IL-22 production by inhibiting FICZ metabolism, CA was first directly compared to FICZ in mouse naïve cell cultures (Fig. 8E). Although FICZ did induce some IL-22 production, IL-22 production was not greater than that caused by CA, even when FICZ was titrated to 10 µM (Fig. 8E, right panel). Next, the ability of CA to inhibit CYP1A1 activity, which would block FICZ metabolism, was tested with a luciferin-based reporter assay. CA was incubated with CYP1A1-loaded microsomes and a CYP1A1 substrate. Dose-dependent inhibition of CYP1A1 activity was seen with CA; however, this inhibition was much less than that caused by known CYP1A1 inhibitors α-napthoflavone and 1-(1-propynyl)pyrene (1-PP) (Fig. 8F). 1-PP in particular has been shown to activate the AHR indirectly through inhibition of CYP1A1-dependent metabolism of an endogenous AHR ligand [37]. Thus, 1-PP was tested in mouse naïve cell cultures to determine whether CYP1A1 inhibition could induce IL-22 in this system. Importantly, concentrations of 1-PP capable of completely inhibiting CYP1A1 were unable to induce IL-22, providing evidence that induction of IL-22 by CA is not the result of CYP1A1 inhibition (Fig. 8G). CA in the presence of 1-PP retained its ability to induce IL-22, showing that 1-PP does not affect the ability of the cells to respond to CA. Therefore, it seems unlikely that CA is exerting its effects through altering FICZ metabolism.

### Generation of CA from immune cells

**Figure 9:**
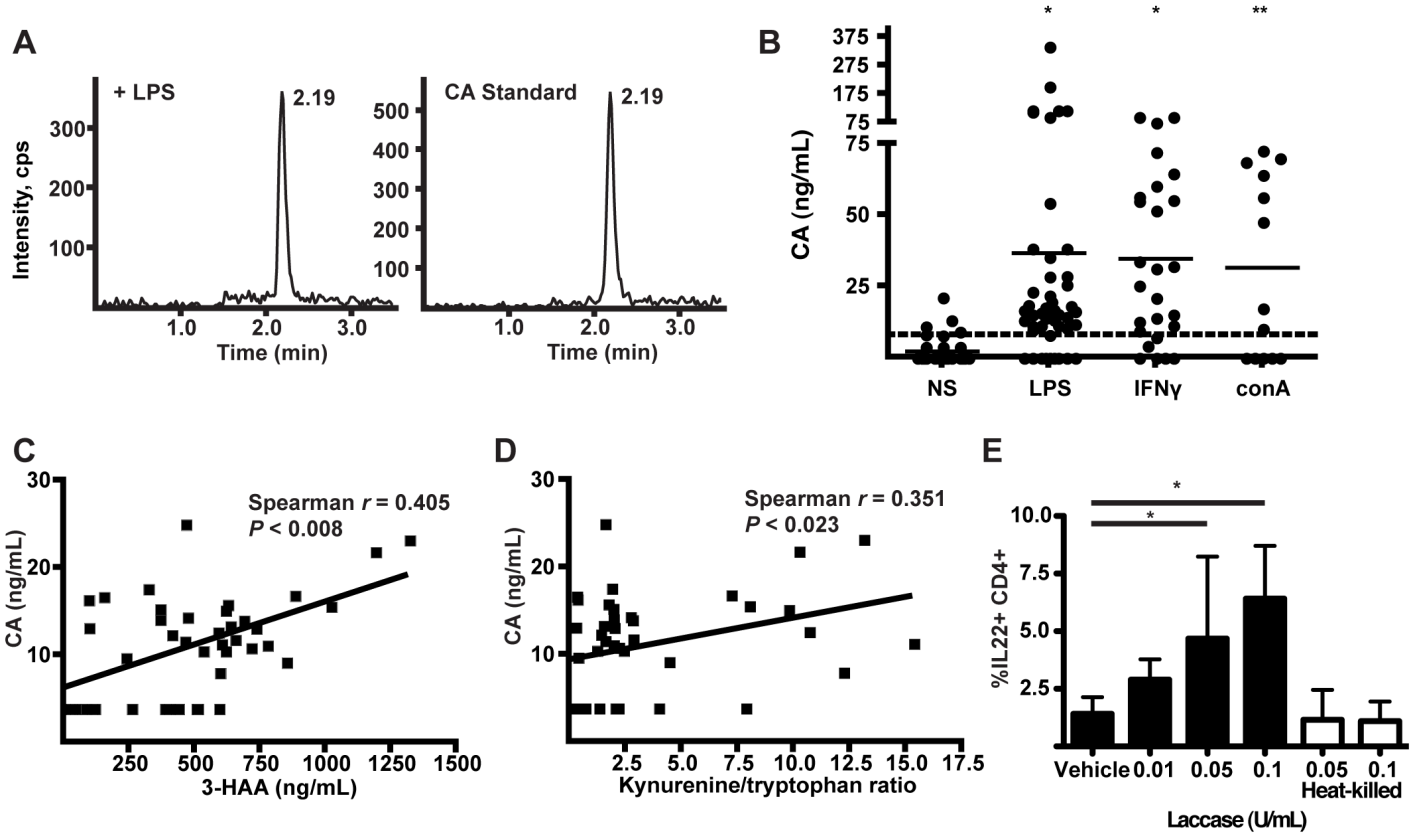
CA is generated by human PBMCs stimulated *in vitro*. (A) Detection of CA by LC/MS/MS in supernatants of human PBMCs stimulated with LPS (50 µg/mL). The left chromatogram is representative of LPS-treated samples, and the right represents a CA standard peak. (B) Measurement of CA in supernatants of human PBMCs cultured with LPS (50 µg/mL), IFNγ (100 ng/mL), or conA (1 µg/mL) for 16 hours versus non-stimulated (NS) controls. Data points represent individual treated wells from human donors. The dashed line represents the limit of quantification (LOQ = 7.81 ng/mL) of CA. Data points below LOQ were treated as ½ LOQ, while data points below the limit of detection (LOD = 3 ng/mL) were treated as zero. Data from different stimulation conditions were compared by one way ANOVA (Kruskal-Wallis, Dunn's Multiple Comparison). *P<0.001, **P<0.01 versus non-stimulated wells. (C) Correlation of the CA concentration in supernatants of human PBMCs with the concentration of 3-HAA (Spearman's rank correlation). Each data point represents an individual sample treated +/− LPS, LPS/PMA, IFNγ, or conA. Samples with CA below LOQ were assigned a value of ½ LOQ. (D) Correlation of CA secretion in supernatants of human PBMCs with the ratio of kynurenine/tryptophan in the supernatant (Spearman's rank correlation). (E) Incubation of sorted naïve mouse CD4^+^ T cells with DMSO, the fungal enzyme laccase, or heat-killed laccase under polarizing conditions (as in panels A, B). Data pooled from four independent experiments were analyzed by one way ANOVA (Kruskal-Wallis with Dunn's Multiple Comparison Test). * p<0.05. Error bars are SD.

We next sought to determine whether immune cells are capable of producing CA. To date, CA has only been identified within human cell cultures in which 3-HAA has been exogenously added [19]. However, the formation of CA *in vivo* in rats injected with LPS has recently been described [38]. We accordingly asked whether human PBMCs are capable of generating CA when stimulated with LPS or other immunostimulatory compounds. After culture for 16 hours, CA at concentrations up to ∼1 µM appeared in the cell culture supernatants of cells stimulated with LPS, IFNγ, or concanavalin A, yet remained low or below the LOQ in unstimulated (NS) control wells (Fig. 9, A and B). Production of CA was correlated with both the endogenous 3-HAA concentration in the supernatant (Fig. 9C) and with the supernatant kynurenine/tryptophan ratio (Fig. 9D), the latter of which is indicative of IDO-driven tryptophan metabolism [30]. To our knowledge, this is the first characterization of CA secretion from human cells *in vitro*. Finally, we tested whether conditions likely to lead to CA generation could affect IL-22 production *in vitro*. The fungal enzyme, laccase, has been described as capable of catalyzing the formation of CA from 3-HAA [39]. When laccase alone was introduced into mouse naïve CD4^+^ T cell cultures under polarizing conditions, IL-22 production was doubled, possibly from formation of CA or a related dimerization product from tryptophan metabolites in the media (Fig. 9E). Laccase that had been heat killed was unable to increase IL-22 production, demonstrating the requirement for its enzymatic activity.

## Discussion

We report here that CA is an endogenous tryptophan metabolite that acts via the AHR to stimulate production of IL-22 in human and mouse lymphocytes. As a downstream metabolite of IDO capable of binding the AHR, CA provides one of the first examples of a chemical mediator from an evolutionarily conserved pathway capable of driving IL-22 production through AHR activation.

The conclusion that CA is acting via the AHR is supported by several lines of evidence, including CA displacement of [^3^H]TCDD from the human AHR, induction of *Cyp1a* in zebrafish embryos in vivo and in human and mouse lymphocytes, AHR-dependent reporter gene induction in H1G1 cells, loss of effects in cells from *Ahr* ^−/−^ mice, and the ability of an AHR antagonist to block the stimulation of IL-22 production by the CA precursor 3-HAA and CA itself in cells from wild-type mice. An alternative interpretation of the results in cells from *Ahr*-null mice is that the lack of AHR affects the differentiation of cells destined to become IL-22-producing CD4^+^ cells and thus interferes with their ability to respond to any inducers of IL-22, regardless of the proximal mechanism involved. In that case, the results in cells from *Ahr*-null mice would be non-informative about the proximal mechanisms that are mediating the effects of CA in cells from wild-type mice. However, the other evidence from AHR binding studies, reporter gene assays, induction of CYP1A1, and inhibition of CA-stimulated IL-22 production by an AHR antagonist all point to a role for the AHR in mediating the effects of CA on IL-22 production.

We have directly compared the ability of CA to induce IL-22 to that of other reported tryptophan-derived AHR agonists (e.g., FICZ, L-KYN and KYA) [15], [16], [40]. Amongst the metabolites downstream of IDO (L-KYN and KYA), the ability of CA to increase IL-22 production from naïve T cells is comparable to that observed with tryptophan photoproduct FICZ (Fig. 8E). Neither L-kynurenine nor kynurenic acid increased IL-22 production as effectively as CA in mouse or human T cells under the tested concentrations and conditions, despite the ability of these two compounds to more effectively induce *CYP1A1*, an AHR-responsive gene, in human and mouse lymphocytes. CA may be a selective AHR modulator (SAhRM) [14], [41], more potently inducing *IL-22* than *CYP1A1*. Indeed, several AHR ligands that bind the AHR and elicit AHR-dependent effects, but that are weak inducers of *CYP1A1*, have been described previously [14], [41]-[44]. The actions of such SAhRMs can be cell- and species-specific [14], [45], and SAhRMs with selective immunomodulatory activity (although not involving IL-22) have been reported previously [46]. The molecular mechanism by which AHR activation leads to enhanced IL-22 expression is not yet well understood, but it appears to involve interaction of AHR with RORγt at the *Il22* gene [47]. Whether this mechanism involves direct DNA binding by the CA-activated AHR or a DNA-binding-independent mechanism (such as tethering to DNA-bound RORγt) [48] remains to be investigated.

In addition to demonstrating that CA is an AHR agonist that promotes IL-22 production, we show that CA can be produced by stimulated human PBMCs in the absence of exogenous 3-HAA. Potential enzymatic modulators that can regulate the generation of CA from 3-HAA would predictably affect the resolution of inflammation. Such enzymes include ceruloplasmin [39], superoxide dismutase [33], catalase [33], and the fungal virulence factor, laccase [39]. It is interesting to note that ceruloplasmin recently has been shown to be protective in mouse models of inflammatory bowel disease, where IL-22 has also been shown to be protective [49], [50]. CA might also be generated through non-enzymatic reactions favored under oxidizing conditions [51], such as those found in the context of inflammatory responses. For instance, neutrophils, which produce reactive oxygen species (ROS) in an antimicrobial oxidative burst, also express high levels of IDO in the setting of fungal infections [52]. In such cells, co-expression of IDO and enzymes involved in generating ROS might skew the tryptophan metabolic pathways towards the generation of CA over PA or QA. Although CA is effective at driving IL-22 production only at the upper limit of secreted concentrations detected in our assays (∼1 µM) (Figs. 8D, 9B), it is important to note that the relationship between concentrations of AHR ligands required for effects *in vitro* versus those required *in vivo* remain unknown. Differential concentrations of serum proteins, for example, within *in vitro* assays may reduce the bioavailability and the apparent potency of AHR agonists [53]. In addition, and as in the case for spatially-regulated secretion of cytokines [54], local concentrations of ligands at points of cell-cell contact within hematolymphoid organs may approximate or even exceed those that can be achieved *in vitro* or be found in the peripheral circulation *in vivo*.

Environmentally generated ligands for the AHR have been recently shown to affect homeostasis between the immune system and commensal microflora in the gut mucosa [55]. AHR activation was found to be critical for maintenance of local intraepithelial lymphocyte (IEL) subsets that in turn regulate the homeostasis of and prevent bacterial dissemination across the mucosal epithelium. In a separate report, innate lymphoid cells producing IL-22 (ILC22) in the gut were also shown to be AHR-dependent [56]. In some of these cases, AHR activation was induced by exogenous ligands; for gastrointestinal immunity, the presumptive AHR ligands were dietary, whereas tryptophan photoproducts such as FICZ may be generated by UV exposure of the skin. By contrast, removal of dietary AHR ligands had no effect on the function of ILC22s [55]. Immune development accordingly appears to be guided by both environmental and endogenous AHR ligands. As in the case of CA, however, the availability, effective concentrations, and tissue distribution of environmentally derived ligands *in vivo* remains to be completely described.

We have shown previously that tryptophan catabolism can result in a loss of Th17 cells in the context of HIV disease through generation of 3-HAA [30]. We hypothesize that this loss, particularly within the gut mucosa, allows for ongoing inflammation due to continued microbial translocation. Conversion of 3-HAA into CA could reverse the effects of 3-HAA within immune cells, and thereby restore IL-22-producing cells in the context of increased IDO activity. This would allow for the resolution of the inflammatory signaling cascade by strengthening the mucosal barrier, thus stopping a vicious cycle that might otherwise drive disease progression [30]. Although IL-22 was initially linked to IL-17 as a pro-inflammatory cytokine, recent evidence suggests that it probably plays an independent immunoregulatory role in the context of non-hematopoietic cells, maintaining epithelial cell homeostasis in the mucosal tissues [50], [57], [58]. If so, the pathways that lead to the generation of CA may operate in tandem with the immunosuppressive mechanisms linked to tryptophan metabolism to generate a population of IL-22 producing cells that plays a specific role in tissue repair following inflammation [58]. These findings prompt future investigation into the potential roles that CA may play in numerous biological settings in which the AHR is involved.

## Supporting Information

Figure S1 **Effects of 3-HAA on IL-22 production from CD4^+^ T cells in total human PBMCs.** Flow cytometric analysis of the frequency of CD4^+^IL22^+^ T cells relative to total events collected (left panel) and average fold change for individual donors relative to DMSO control (right panel) following stimulation of PBMCs in the presence of increasing concentrations of 3-HAA (µM) for six days. Error bars indicate SD. Data were analyzed by one-way ANOVA with Dunnett's multiple comparisons test (left panel) and one sample t test comparing to a theoretical mean of 1 (right panel). *, p<0.05; **, p<0.01.
(TIF)

Figure S2 **Effects of CA on IL-22 production from CD4^+^ T cells in total human PBMCs.** Fold change in frequency of IL-22 CD4^+^ T cells relative to total events from human PBMCs from multiple donors stimulated in the presence of CA versus DMSO control. Data were analyzed by one sample t test for significant deviation from a theoretical mean of 1.000. *p<0.05. Error bars are SD.
(TIF)

Figure S3 **Effects of CA on cytokine production from human CD4^++^ T cells in total naïve cell cultures.** Flow cytometric analysis of the frequency of CD4^+^IL22^+^ T cells relative to total events collected from sorted naïve human CD4^+^ T cells stimulated under polarizing conditions (with IL-21, IL-1β, IL-23, anti-IFNγ, anti-IL-4, and anti-IL12) with DMSO, 3-HAA (25 µM), or CA (25 µM). Data on IL-22, IFNγ, and IL-17 production are from three independent experiments. Error bars are SD.
(TIF)

Figure S4 **Effects of CA on human Treg differentiation in total naïve cell cultures.** Quantification of %FOXP3^+^CD25^+^ T cells of total events from naïve CD4^+^ T cells stimulated in the presence of CA (10 or 25 µM) or DMSO with increasing concentrations of TGF-β. Data from six donors in seven independent experiments were analyzed by two-way ANOVA and Holm-Sidak's multiple comparisons test. *, p<0.05. Error bars are SD.
(TIF)

Figure S5 **Effects of CA on cytokine production from mouse CD4^+^ T cells in total naïve cell cultures.** Flow cytometric analysis of the frequency of IL22^+^ (left) and IL17^+^ (right) CD4^+^ T cells relative to total events from sorted naïve mouse CD4^+^ T cells from C57BL/6 mice stimulated under polarizing conditions (with IL-1β, IL-6, TGF-β, anti-IFNγ, and anti-IL12/23) in the presence of CA (25 or 35 µM) or DMSO (DMSO controls for the 25 and 35 µM CA experiments are shown separately). Data from six independent experiments were analyzed by one-way ANOVA and Bonferroni's multiple comparisons test. *, p<0.05. Error bars are SD.
(TIF)

Figure S6 **Effects of CA on mouse Treg differentiation in total naïve cell cultures.** Flow cytometric analysis of frequency of FOXP3^+^CD25^+^ CD4^+^ T cells relative to total events from sorted naïve wild-type mouse CD4^+^ T cells stimulated with increasing concentrations of TGFβ in the presence of DMSO or CA (10 or 25 µM). Quantification of %FOXP3^+^CD25^+^ T cells of CD4^+^ cells from four independent experiments is shown. Error bars are SD.
(TIF)

Figure S7 **Effects of tryptophan metabolites on IL-22 production from human CD4^+^ T cells in total naïve cell cultures.** Flow cytometric analysis of human cord blood naïve CD4^+^ T cells stimulated under polarizing conditions (IL-1β, IL-6, IL-23, TGF-β, anti-IFNγ, and anti-IL4) with DMSO, CA, L-KYN, or KYA (concentrations in µM). Frequency of IL22^+^ CD4^+^ T cells relative to total events from eight experiments was analyzed by one-way ANOVA and Dunnett's Multiple Comparisons Test. *, P<0.05.
(TIF)

Figure S8 **Dose-response effect of CA on IL-22 production from human CD4^+^ T cells in total naïve cell cultures.** Flow cytometric analysis of cord blood naïve CD4^+^ T cells stimulated under polarizing conditions (as in Figure S7) with DMSO or CA (0.5, 1, 5, or 10 µM). Frequency of IL22^+^ CD4^+^ T cells relative to total events from four experiments was analyzed by repeated measures one-way ANOVA and Dunnett's Multiple Comparisons Test. *, p<0.05.
(TIF)
