# Supplementary figures and images for: Identification of Cinnabarinic Acid as a Novel Endogenous Aryl Hydrocarbon Receptor Ligand That Drives IL-22 Production
Source: PLoS One. 2014 Feb 3;9(2):e87877. doi: 10.1371/journal.pone.0087877 (PMC3912126; doi:10.1371/journal.pone.0087877)

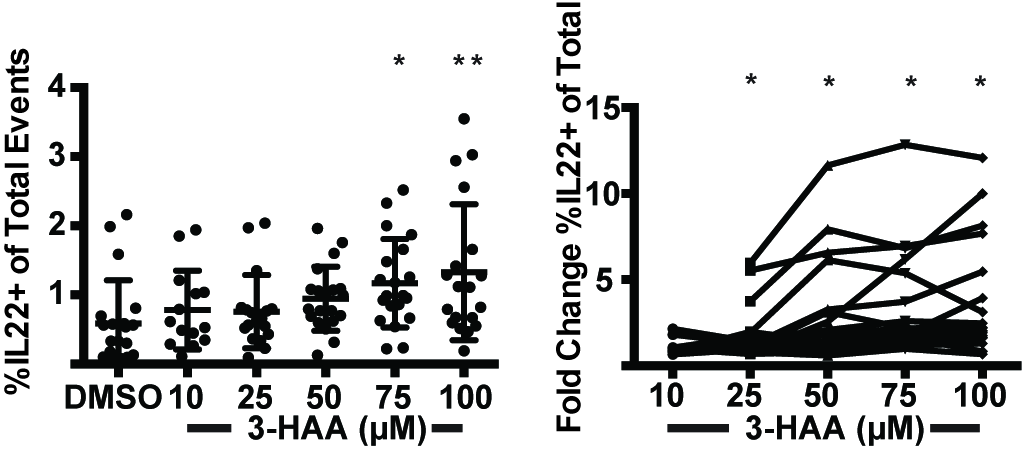

Supplement: Figure S1 — Effects of 3-HAA on IL-22 production from CD4+ T cells in total human PBMCs. Flow cytometric analysis of the frequency of CD4+IL22+ T cells relative to total events collected (left panel) and average fold change for individual donors relative to DMSO control (right panel) following stimulation of PBMCs in the presence of increasing concentrations of 3-HAA (µM) for six days. Error bars indicate SD. Data were analyzed by one-way ANOVA with Dunnett's multiple comparisons test (left panel) and one sample t test comparing to a theoretical mean of 1 (right panel). *, p<0.05; **, p<0.01. (TIF) [file pone.0087877.s001.tif]

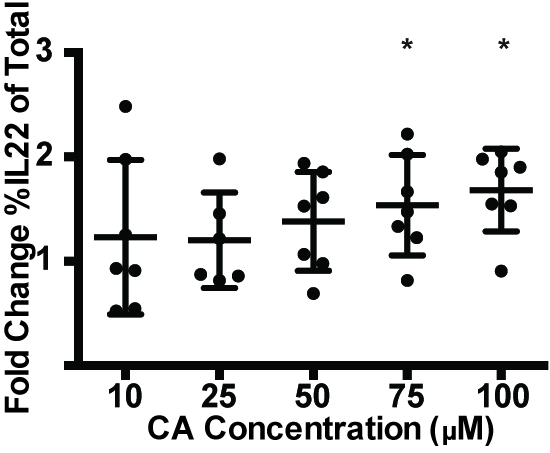

Supplement: Figure S2 — Effects of CA on IL-22 production from CD4+ T cells in total human PBMCs. Fold change in frequency of IL-22 CD4+ T cells relative to total events from human PBMCs from multiple donors stimulated in the presence of CA versus DMSO control. Data were analyzed by one sample t test for significant deviation from a theoretical mean of 1.000. *p<0.05. Error bars are SD. (TIF) [file pone.0087877.s002.tif]

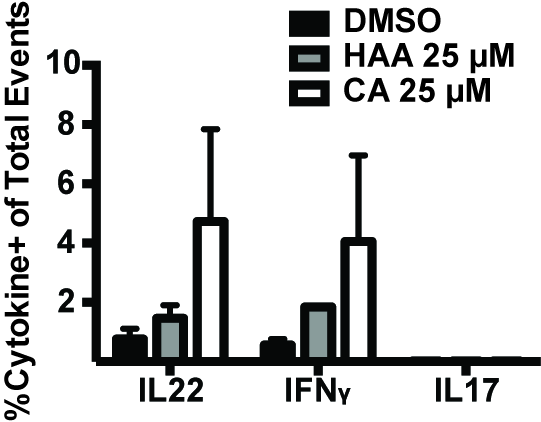

Supplement: Figure S3 — Effects of CA on cytokine production from human CD4++ T cells in total naïve cell cultures. Flow cytometric analysis of the frequency of CD4+IL22+ T cells relative to total events collected from sorted naïve human CD4+ T cells stimulated under polarizing conditions (with IL-21, IL-1β, IL-23, anti-IFNγ, anti-IL-4, and anti-IL12) with DMSO, 3-HAA (25 µM), or CA (25 µM). Data on IL-22, IFNγ, and IL-17 production are from three independent experiments. Error bars are SD. (TIF) [file pone.0087877.s003.tif]

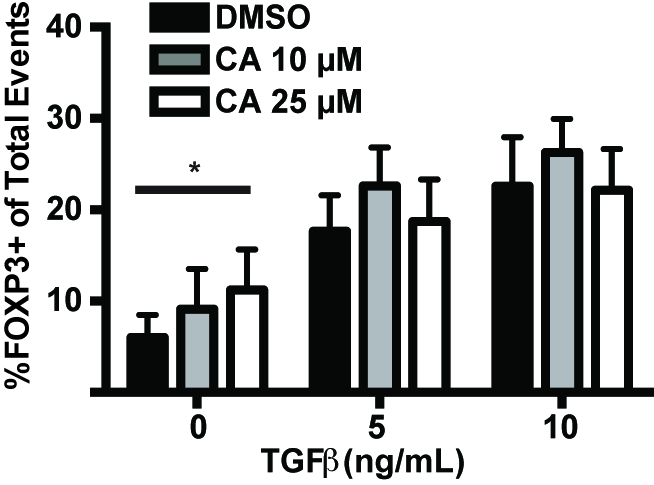

Supplement: Figure S4 — Effects of CA on human Treg differentiation in total naïve cell cultures. Quantification of %FOXP3+CD25+ T cells of total events from naïve CD4+ T cells stimulated in the presence of CA (10 or 25 µM) or DMSO with increasing concentrations of TGF-β. Data from six donors in seven independent experiments were analyzed by two-way ANOVA and Holm-Sidak's multiple comparisons test. *, p<0.05. Error bars are SD. (TIF) [file pone.0087877.s004.tif]

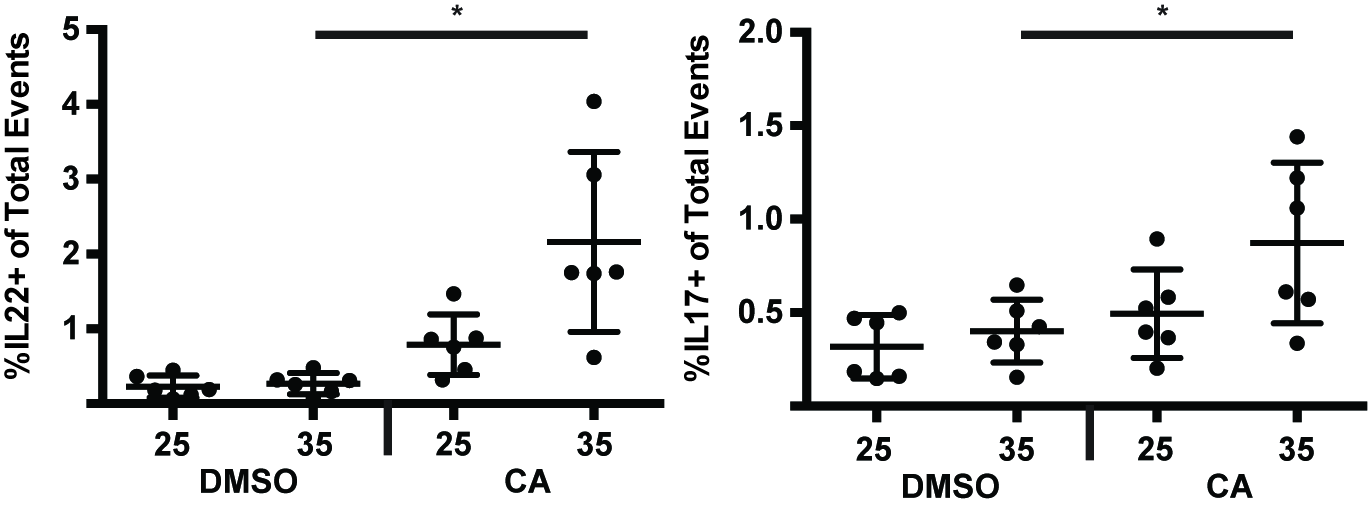

Supplement: Figure S5 — Effects of CA on cytokine production from mouse CD4+ T cells in total naïve cell cultures. Flow cytometric analysis of the frequency of IL22+ (left) and IL17+ (right) CD4+ T cells relative to total events from sorted naïve mouse CD4+ T cells from C57BL/6 mice stimulated under polarizing conditions (with IL-1β, IL-6, TGF-β, anti-IFNγ, and anti-IL12/23) in the presence of CA (25 or 35 µM) or DMSO (DMSO controls for the 25 and 35 µM CA experiments are shown separately). Data from six independent experiments were analyzed by one-way ANOVA and Bonferroni's multiple comparisons test. *, p<0.05. Error bars are SD. (TIF) [file pone.0087877.s005.tif]

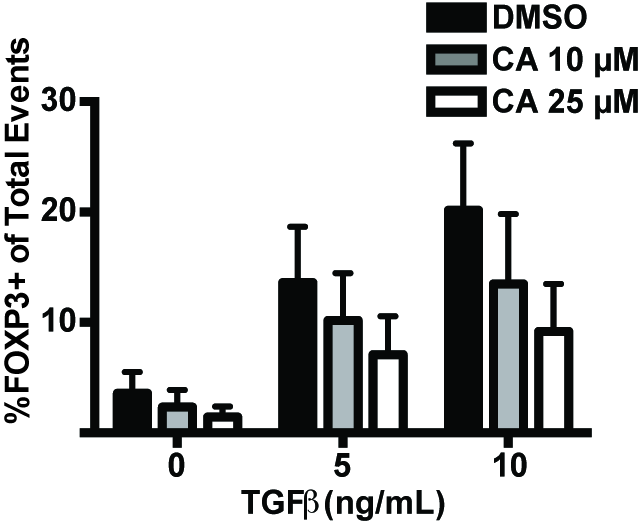

Supplement: Figure S6 — Effects of CA on mouse Treg differentiation in total naïve cell cultures. Flow cytometric analysis of frequency of FOXP3+CD25+ CD4+ T cells relative to total events from sorted naïve wild-type mouse CD4+ T cells stimulated with increasing concentrations of TGFβ in the presence of DMSO or CA (10 or 25 µM). Quantification of %FOXP3+CD25+ T cells of CD4+ cells from four independent experiments is shown. Error bars are SD. (TIF) [file pone.0087877.s006.tif]

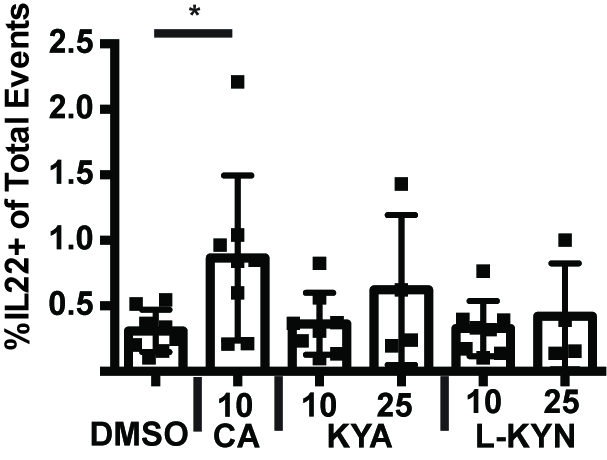

Supplement: Figure S7 — Effects of tryptophan metabolites on IL-22 production from human CD4+ T cells in total naïve cell cultures. Flow cytometric analysis of human cord blood naïve CD4+ T cells stimulated under polarizing conditions (IL-1β, IL-6, IL-23, TGF-β, anti-IFNγ, and anti-IL4) with DMSO, CA, L-KYN, or KYA (concentrations in µM). Frequency of IL22+ CD4+ T cells relative to total events from eight experiments was analyzed by one-way ANOVA and Dunnett's Multiple Comparisons Test. *, P<0.05. (TIF) [file pone.0087877.s007.tif]

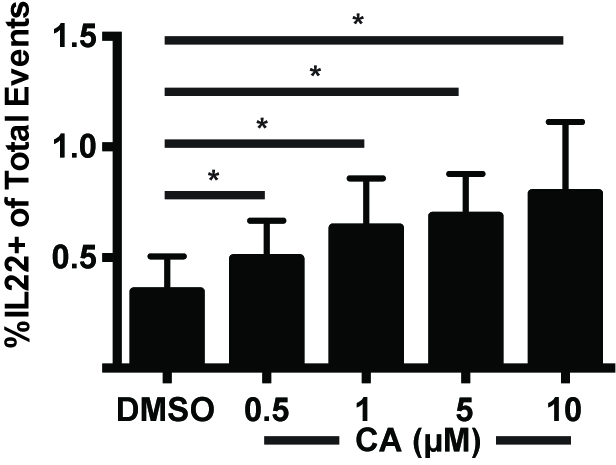

Supplement: Figure S8 — Dose-response effect of CA on IL-22 production from human CD4+ T cells in total naïve cell cultures. Flow cytometric analysis of cord blood naïve CD4+ T cells stimulated under polarizing conditions (as in Figure S7) with DMSO or CA (0.5, 1, 5, or 10 µM). Frequency of IL22+ CD4+ T cells relative to total events from four experiments was analyzed by repeated measures one-way ANOVA and Dunnett's Multiple Comparisons Test. *, p<0.05. (TIF) [file pone.0087877.s008.tif]
